# Influence of Epoxidized Canola Oil (eCO) and Cellulose Nanocrystals (CNCs) on the Mechanical and Thermal Properties of Polyhydroxybutyrate (PHB)—Poly(lactic acid) (PLA) Blends

**DOI:** 10.3390/polym11060933

**Published:** 2019-05-29

**Authors:** Adrián Lopera-Valle, Joseph V. Caputo, Rosineide Leão, Dominic Sauvageau, Sandra Maria Luz, Anastasia Elias

**Affiliations:** 1 Donadeo Innovation Centre for Engineering, Department of Chemical and Materials Engineering, University of Alberta, Edmonton, AB T6G 1H9, Canada; lopera@ualberta.ca (A.L.-V.); jcaputo@ualberta.ca (J.V.C.); dominic.sauvageau@ualberta.ca (D.S.); sandra.unb@gmail.com (S.M.L.); 2 Department of Automotive Engineering, University of Brasília, Faculdade do Gama, Brasília-DF 72444-240, Brazil; rosemirandaleao@gmail.com

**Keywords:** cellulose nanocrystals (CNCs), epoxidized canola oil (eCO), mechanical properties, polyhydroxyalkanoate (PHA), polyhydroxybutyrate (PHB), poly(lactic acid) (PLA), thermal properties

## Abstract

Two major obstacles to utilizing polyhydroxybutyrate (PHB)—a biodegradable and biocompatible polymer—in commercial applications are its low tensile yield strength (<10 MPa) and elongation at break (~5%). In this work, we investigated the modification of the mechanical properties of PHB through the use of a variety of bio-derived additives. Poly(lactic acid) (PLA) and sugarcane-sourced cellulose nanocrystals (CNCs) were proposed as mechanical reinforcing elements, and epoxidized canola oil (eCO) was utilized as a green plasticizer. Zinc acetate was added to PHB and PLA blends in order to improve blending. Composites were mixed in a micro-extruder, and the resulting filaments were molded into 2-mm sheets utilizing a hot-press prior to characterization. The inclusion of the various additives was found to influence the crystallization process of PHB without affecting thermal stability. In general, the addition of PLA and, to a lesser degree, CNCs, resulted in an increase in the Young’s modulus of the material, while the addition of eCO improved the strain at break. Overall, samples containing eCO and PLA (at concentrations of 10 wt %, and 25 wt %, respectively) demonstrated the best mechanical properties in terms of Young’s modulus, tensile strength and strain at break.

## 1. Introduction

While petroleum-based polymers exhibit excellent properties and are low in cost, the negative environmental effects resulting from their use continues to grow. Biopolymers—derived from plants or bacteria (using renewable resources as sources of carbon)—are attractive alternatives. In addition, some biopolymers are degradable, which can reduce the accumulation of plastics in the environment after disposal [1,2]. Polyhydroxybutyrate (PHB)—part of the polyhydroxyalkanoate (PHA) aliphatic polyester class—is a semi-crystalline polymer produced through numerous mechanisms, including the bacterial fermentation of sugars. PHB can be enzymatically-degraded by microorganism found in a variety of environments [3]. These microorganisms produce enzymes (PHA depolymerases) that catalyze the hydrolysis of the polymer, yielding monomers and oligomers that can then be consumed by the microorganisms. PHB—as well as its degradation products—are both food safe and biocompatible, making it a good candidate for applications ranging from food packaging to pharmaceuticals [4,5,6,7,8]. While the tensile strength of PHB is comparable to that of the semi-crystalline synthetic polymer polypropylene (~30 MPa), it has both a low tensile yield strength (<10 MPa) and low elongation at break (<5%) [9,10]. These drawbacks have led to the need to develop methods in order to improve the mechanical properties of PHB without compromising other properties, such as thermal stability.

The mechanical and thermal properties of biopolymers can be tailored through the addition of plasticizers [11,12,13]. However, these are often non-biodegradable and may even be toxic to the environment and/or human health, which leads to negative effects when released into ecosystems [14,15]. There is thus strong incentives and interest in using plasticizers that are both biodegradable and biocompatible. In the literature, various epoxidized oils (soybean oil, linseed oil, etc.) have been utilized as plasticizers of the bio-polymers PHB [12], poly(3-hydroxybutyrate-*co*-3-hydroxyvalerate) PHBV [13], and poly(lactic acid) (PLA) [11]. These oils are generally triglycerides comprised of mono- or polyunsaturated fatty acids. The double bond of the fatty acid can be functionalized with an epoxy group to increase reactivity and reduce migration of the oil through the material. For instance, Garcia-Garcia et al. [16] studied the effects of different concentrations of epoxidized linseed oil (ELO) and epoxidized soybean oil (ESBO) on the mechanical and thermal properties of PHB. Their results showed that adding the epoxidized oils led to a decrease in ultimate tensile stress from 24 MPa for neat PHB to 15 MPa for 20 wt % of ELO and ESBO. Moreover, the addition of 5 wt % and 10 wt % of ELO led to an increase in strain at break of 4.5% and 5%, respectively. Differences between the effects of the different oils can be attributed to differences in the content of unsaturated, monosaturated, and polysaturated fatty acids that comprise the triglycerides. As epoxide groups are functionalized on C-C double bonds in the chain of the fatty acid, a higher degree of saturation enables the addition of a higher number of epoxide groups. One lesser explored material is epoxidized canola oil (eCO) [17]. While canola oil is lower in polyunsaturated fatty acids than soybean oil, it is high in monosaturated oleic acid (~61%) and low in saturated fatty acids, which means that it has a reasonably high number of double bonds available for epoxidation. This particular epoxidized oil has not been previously examined for use as a plasticizer for PHB. 

The addition of eCO is expected to efficiently increase the elongation at break of PHB, while decreasing its elastic modulus and tensile strength. Moreover, the mechanical properties of PHB can be improved by forming blends with other biopolymers [18,19]. In particular, PHB has been blended with poly(lactic acid) (PLA), which is a biosourced recyclable thermoplastic that has received high levels of commercial attention. PLA is obtained from ring-opening polymerization of the monomer lactide, a product of depolymerisation of lactic acid which is sourced from the fermentation of sugarcane, sugar beet, and corn. PLA has a higher storage modulus, tensile yield strength, elastic modulus, and flexural yield strength than PHB [9,18,20,21]. However, the biological degradation of PLA occurs at a much slower rate than that of PHB, even under ideal conditions (i.e., temperature, pH, humidity etc.) [18,20].

Given that PHB and PLA exhibit different advantageous properties, previous studies have examined blending these polymers, aiming to engineer a material with properties that include higher tensile strength, elongation at break, and thermal stability, faster degradation, and tunable crystallinity [9,22,23,24,25]. For example, Zhang et al. [9] investigated the properties of a melt blended polymer containing different ratios of PHB and PLA, and showed that while PLA/PHB blends are immiscible, they have molecular-level interactions that depend on the molecular weight of the polymers [9]. Additionally, they found that the mixing of PHB with PLA increases the thermal stability of the PHB phase within the polymer blend when compared with neat PHB; improvements in mechanical properties over PLA were seen in a 3:1 blend of PLA/PHB. They attributed these improved properties to the ability of PHB crystals to act as reinforcing agents within the PLA matrix [9]. 

Additional agents may be used to improve the properties of PHB/PLA blends. For example, Xiuyu et al. [26] added a solubilizing agent—poly (butyrate adipate terephthalate) (PBAT, otherwise known as polybutyrate)—to PHB/PLA blends, and found that the miscibility between PHB and PLA was improved [24]. The mechanical characterization of PHB/PLA blends suggested that the addition of PBAT to the system led to a reduction in the Young’s modulus (*E)* and ultimate tensile strength (σ_UTS_), and an increase in elongation at break (ε_b_) [24]. Furthermore, they conducted soil degradation studies and found that the rate of mass loss of PHB/PLA blends solubilized with PBAT was greater than those of either neat PHB or PLA, and increased with increasing content of PBAT [24]. Alternatively, the use of catalysts has also been shown to impact PHA properties. Zinc acetate is known to help catalyze the transesterification reaction between different sets of thermoplastic polymers [26,27]. Yang et al. [26] proposed using zinc acetate as a compatibilizer for the melt blending process of PHBV (poly(3-hydroxybutyrate-*co*-3-hydroxyvalerate), another member of the PHA family, and PLA in a 30:70 ratio. They demonstrated that the miscibility of the PHBV and PLA polymer blend was improved by the introduction of the zinc acetate additive. In addition, they showed that the inclusion of the compatibilizer significantly improved the elongation at break of the blend [26]. 

Cellulose nanocrystals (CNCs)—which can be obtained from an assortment of biomass products such as coconut fibers and wood—have been previously used as reinforcing agents for biopolymers such as PHB, PLA and poly (vinyl alcohol) (PVA), improving their mechanical properties [22,28]. These materials are highly abundant in nature, eco-friendly, and renewable [22,28,29]. Arrieta et al. [22] investigated hot-pressed PLA and PLA-PHB blended films (3:1 ratio) with the addition of 5 wt % pristine CNCs material [22]. In their work, it was found that this addition of 5 wt % of CNCs to neat PLA films led to an increase in both Young’s modulus, (from 1200 MPa to 1500 MPa), and in tensile strength (from 47 MPa to 56 MPa). They concluded that PHB and well-dispersed CNCs were good reinforcing agents that increased simultaneously the elongation at break and Young’s modulus of the polymer films.

When additives are combined, synergistic effects can occur. One purpose of this study is to determine if both the elongation at break and tensile strength of PHB can be improved by blending with eCO and PLA. In addition, we explore if further improvement to these properties can be achieved by adding CNCs to neat PHB and PHB/PLA/eCO blends. In these composites, eCO was expected to acts as a plasticizing agent [11], and it was therefore added at concentrations of 5 and 10 wt %, based on previous work by Garcia-Garcia et al. [16]. Blending with PLA was expected to produce a material with improved stiffness and strength. In our work, a 3:1 ratio of PHB:PLA was selected as it is the lowest content of PLA in PHB previously reported to improve mechanical properties [9]. To further improve the blending between the two polymers, 0.4 wt % zinc acetate was added as a compatibilizer [26,27]. Finally, CNCs were explored as additional reinforcing filler, and were added to both neat PHB and PHB/PLA/eCO blends. Based on the previous work of Arrieta et al., CNCs were added at 5 wt % and 10 wt % [29]. The individual and synergistic effects of these blends and additives were studied. Both the mechanical properties and the morphologies of the resulting materials were characterized. As ideally the addition of fillers should not reduce the thermal stability of the material, thermal analysis of the samples was also performed.

## 2. Materials and Methods 

### 2.1. Materials

Thermally processed polyhydroxybutyrate (PHB, measured Mw: 190 kDa, more details in supplementary information of this manuscript, BRS Bulk Bio-Pellets, Bulk Reef Supply, Golden Valley, MN, USA) and poly(lactic acid) (PLA, nominal Mw: 390 kDa, 4043D pellets, NatureWorks LCC, Minnetonka, MN, USA) pellets were used. X-ray photoelectron spectroscopy (XPS) surface analysis was used to measure the chemical composition of the as-received pellets and it was found that their surface contained less than 5 wt % ± 2.1 wt % (*n =* 5) calcium (Ca), sodium (Na), and silicon (Si), which likely remained as impurities after the pelletization process. Epoxidized canola oil (eCO) was synthetized following methods described by Mungroo et al. [17]. Fourier transform infrared (FTIR) spectroscopy measurements were performed on canola oil (CO) and epoxidized canola oil (eCO) to confirm epoxidation took place. Further information on the FTIR method and results (Figure S1) are included in the supplementary information of this manuscript. Zinc acetate crystalline powder (Cat, No. 383317, Sigma Aldrich, St. Louis, MO, USA) was used as received.

Cellulose nanocrystals (CNCs) were prepared from sugarcane bagasse, as reported by Leão et al. [28]. Described as condition IX in their study, the sugarcane bagasse was pre-treated in a 3.3 w/v % solution of sodium chlorite (NaClO_2_), with 10 drops of acetic acid (C_2_H_4_O_2_) at 90 °C for 1 h in order to break the lignocellulosic structure and to remove lignin and hemicellulose. Following this, the pre-treated bagasse was bleached with a 5 w/v % solution of sodium hydroxide (NaOH) at 80 °C for 1 h, and subsequently in a 1 M solution of nitric acid (HNO_3_) at 90 °C for 1 h. The CNCs were obtained by hydrolysis of the bleached pulps using a 64 wt % solution of sulfuric acid (H_2_SO_4_) at 45 °C for 0.5 h. Finally, cellulose was repeatedly washed and filtered by centrifugation at 7830 rpm for 20 min. A transmission electron microscope (TEM JEM 2100, JOEL Ltd., Peabody, MA, USA) was used to measure the dimensions of the CNCs, and an X-ray diffractometer (XRD, Ultima IV, Rikagu Co., Woodlands, TX, USA) was used to estimate their crystallinity. The length, diameter and crystallinity index of the CNCs was 220 nm, 12 nm and 65%, respectively [28]. Further information on the TEM and XRD methods and results (Figure S2) are included in the supplementary information of this manuscript.

### 2.2. Sample Preparation

The PHB and PLA pellets were washed with isopropyl alcohol to remove surface contaminants and dried at 70 °C for at least 8 h prior to use. Following the drying process, the polymers, epoxidized canola oil, and/or CNCs were mechanically blended in 50-mL centrifuge tubes using a vortex mixer (MX-S, SCI-LOGEX, Rocky Hill, CT, USA) for as long as 5 min. Prior to mixing with eCO and/or CNCs, those compositions containing PHB and PLA mixtures were mechanically mixed with 0.4 wt % zinc acetate solution (0.01 g/mL in methanol) in a 400-mL beaker for at least one hour in order to completely dry the methanol. Table 1 lists the contents of PHB, PLA, eCO, and CNCs in the compositions tested, hereafter referenced to as listed in the “Composition” column. The melt-mixing of the samples was carried out using a conical twin-screw micro-extruder (HAAKE™ Minilab II micro compounder, Thermo Fisher Scientific, Waltham, MA, USA), at a temperature of 175 °C and extrusion screw speed of 35 RPM with a residence mixing time of approximately 5 min. These parameters were chosen to obtain void-free pellet-like materials while preventing thermomechanical degradation [30]. 

Following extrusion, the material was pressed using a hydraulic hot-press (No. 4386, Carver Inc., Wabash, IN, USA). A three-part AISI 1008 steel mold was used to make polymer sheets from the extruded material. The bottom and top parts of the mold consisted of flat steel sheets, while the middle part was a 2-mm thick steel sheet with a rectangular 70 mm × 80 mm cut-out. The bottom and middle sheets of the mold were placed on the bottom platen of the hydraulic hot-press, and approximately 5 g of polymer pellets were placed in the rectangular section of the mold (heated to 175 °C), and allowed to soften for 1.5 min. The top plate of the mold was gently placed on top. After this time, light pressure was applied for 30 s to allow the material to spread throughout the middle part of the mold. Finally, a pressure of 2.1 MPa was applied for 1 min. The pressure was then released, and the sample and mold were removed from the press and allowed to cool at ambient temperature. Following pressing, the resulting sheets were pressed once more into steel molds cut with dog-bone and rectangular shapes with appropriate dimensions for tensile testing and dynamic mechanical analysis (DMA) (Section 2.4 Mechanical Characterization). In our previous work, we noticed that the crystallinity of PHB samples tends to increase in the first few days after processing; the specimens were therefore aged at room temperature for seven days prior to characterization. 

### 2.3. Morphology

The morphology of the failure surface of the tensile tested samples was observed using a field emission scanning electron microscope (FE-SEM) (Zeiss Sigma 300 VP-FESEM, Zeiss, Cambridge, UK). Cross-sectional samples were cut from the fractured surfaces of the dog-bone samples after tensile testing was completed. To reduce surface charging during SEM imaging, a thin film of carbon was deposited onto the samples using a carbon evaporation system (EM SCD 005, Leica Baltec Instruments, Balzers, Liechtenstein). The images were captured using secondary electron (SE) mode with a voltage of 5.00 kV. 

### 2.4. Mechanical Characterization

A tensile test machine (Instron 5943, Instron, Norwood, MA, USA) equipped with a 1 kN load cell was used to perform tension mechanical testing on the hot-pressed samples. As per ISO Standard 527-2, a strain rate of 2 mm/min was applied to type 5A bone-shaped flat specimens, with total length, gauge length, gauge width and thickness of 76 mm, 20 mm, 4 mm and 2 mm, respectively [31,32]. The Young’s modulus (E), yield stress (σy), strain at yield (εy), ultimate tensile strength (σUTS) and strain at break (εb) values were determined. Six (n = 6) samples of each type were characterized, and average values and standard deviations were determined and reported.

In order to compare the results from tensile test, dynamic mechanical properties of the polymer blends were tested using a dynamic mechanical analyzer (DMA 8000, PerkinElmer, Inc., Waltham, MA, USA). The flat specimens (12.5 mm in total length, 6.8 mm in gauge length, 5 mm in width, and 2 mm in thickness) were heated from 20 to 150 °C. The tests were performed in single cantilever mode at a 1 Hz frequency, 0.05 mm displacement, and a heating rate of 3 °C/min.

### 2.5. Thermal Characterization

Thermal analysis was performed seven days after hot-pressing (see Section 2.2 Sample Preparation) [33], using a differential scanning calorimeter (DSC, DSC Model 1, Mettler Toledo, Columbus, OH, USA). Samples with mass of ~15 mg were cut and placed in unsealed aluminum pans (n = 3). Analysis was conducted under a nitrogen flow of 50 mL/min, and consisted of a heating cycle from −20 °C to 210 °C at 10 °C/min, followed by an isothermal hold at 210 °C for 5 min, and concluding with a cooling cycle from 210 °C to −20 °C, at a cooling rate of 20 °C/min. These heating/cooling rates were chosen in order to limit the extent of recrystallization and thermal effects during the heating cycle [5]. From the DSC heating scans, glass transition temperature ($T_{g}$), recrystallization temperature ($T_{C}$), melting temperature ($T_{m}$), and enthalpy of melting ($\Delta H_{m}$) were determined. The degree of crystallinity of PHB ($X_{C,\;PHB}$) was determined as a function of melting peak area using Equation (1):

(1) $$X_{c,\;PHB}\;\left(\%\right)=\;\frac{\Delta H_{m}}{\Delta H_{m}^{0}\cdot w}\times 100$$

where, $\Delta H_{m}^{0}$ is the melting heat associated with pure crystalline material (146 J/g for PHB and 96 J/g for PLA), and w is the weight fraction of the PHB in the blend [12,34,35,36]. The $\Delta H_{m}$ was measured from the DSC using an integral tangential baseline setting in the Mettler Toledo STARe thermal analysis software (Mettler Toledo, Columbus, OH, USA).

The thermal stability of the polymer blends was studied by using a thermogravimetric analyzer (TGA/DSC Model 1, Mettler Toledo, Columbus, OH, USA) equipped with the ultra-micro balance cell and differential thermal analysis (DTA) sensors. Samples of ~15 mg were cut from hot-pressed sheets and aged 7 days prior to characterization. Scans were carried out from 25–380 °C at a heating rate of 10 °C/min under 20 mL/min nitrogen atmosphere.

### 2.6. Statistical Analysis

T-test analysis was performed using commercial software (STATISTICA for Academia, StatSoft Inc., Tulsa, OK, USA) in order to evaluate the statistical differences between sets of results for mechanical and thermal testing. Unequal variances and a significance level of α = 0.05 were used in the evaluations, corresponding to a 95% confidence interval.

## 3. Results

### 3.1. Morphology

**Figure 1:**
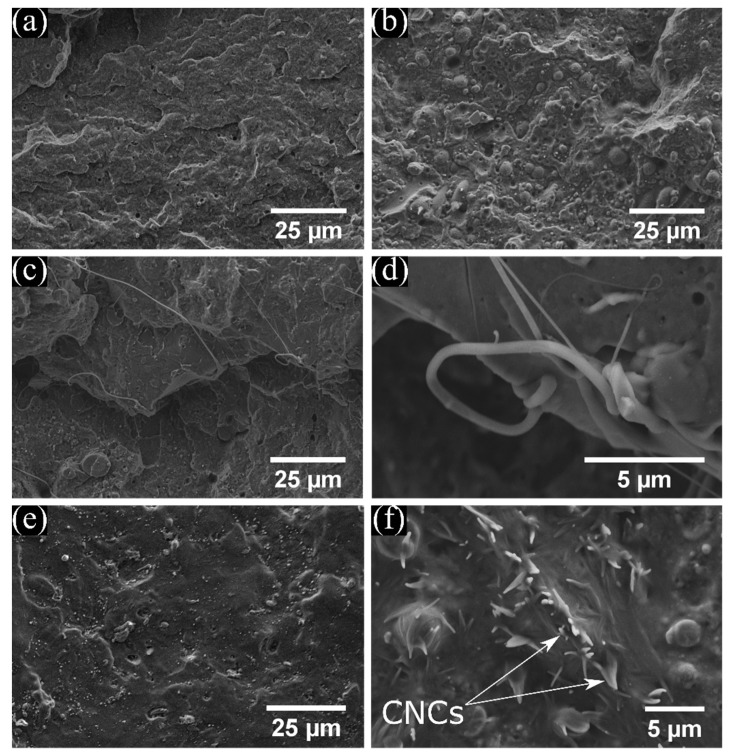
SEM micrographs of failure surfaces for (**a**) neat PHB, (**b**) PHB/10eCO, (**c**) PHB/PLA, (**d**) high magnification of poly(lactide acid) (PLA) phase in PHB/PLA composition, (**e**) PHB/5CNC, and (**f**) high magnification of cellulose nanocrystals (CNCs) in PHB/5CNC composition.

SEM images were collected to analyze the fracture surfaces of tensile test samples. Figure 1 shows representative images of the surface failure of neat PHB, PHB/10eCO, PHB/PLA, and PHB/5CNC. The smooth appearance of the fractured surface of the neat PHB sample (Figure 1a) suggests it underwent brittle failure. On the other hand, PHB and PHB/PLA samples containing eCO (e.g., PHB/10eCO in Figure 1b) exhibit rough surfaces with more protruding features after deformation, suggesting these samples underwent plastic deformation prior to fracture. Figure 1c shows the failure surface of a PHB/PLA sample. In this image, the sample shows signs of a brittle fracture, characterized by a smooth surface with no noticeable plastic deformation (as shown by the lack of dimple-like structures). The images of compositions containing PLA consistently showed the presence of fiber-like structures on the surface of the failure (although no fibers were added). A representative high magnification image of these features is shown in Figure 1d. The presence of these fiber-like structures was observed exclusively in compositions containing PLA, suggesting part of the PLA pellets that were melted were largely deformed into fiber-like structures inside the PHB matrix. Similar results were observed by D’Amico et al. [37], who examined the microstructure of the failure surfaces of 1:1 PHB/PLA blends, and attributed the observed structures to the limited miscibility of the materials.

In the compositions containing eCO (Figure 1b), some voids were observed. These voids could be associated with the miscibility and inefficient mixing between of eCO and PHB. This could be caused by the triglycerides of eCO forming clusters within the amorphous regions of the polymer. Özgür-Seydibeyoǧlu et al. [13] reported similar observations around the failure surface of polyhydroxybutyrate-co-valerate (PHBV) when epoxidized soybean oil was blended [13]. 

The failure surface of PHB/5CNC (Figure 1e) and PHB/PLA/10eCO/5CNC (results not shown) exhibited similar smooth features to those of neat PHB, thus also suggesting brittle failure. Finally, images of PHB/5CNC (Figure 1e,f) and PHB/PLA/10eCO/5CNC compositions showed isolated clusters of CNCs were exposed on the failure surface. Similar structures have been previously reported in PHB and PLA composites by Gan et al. [38] and López de Dicastillo et al. [39], respectively. In their work, an agglomeration of CNCs were found on the surface of the brittle failure of PHB and PLA composites. 

### 3.2. Mechanical Properties

**Figure 2:**
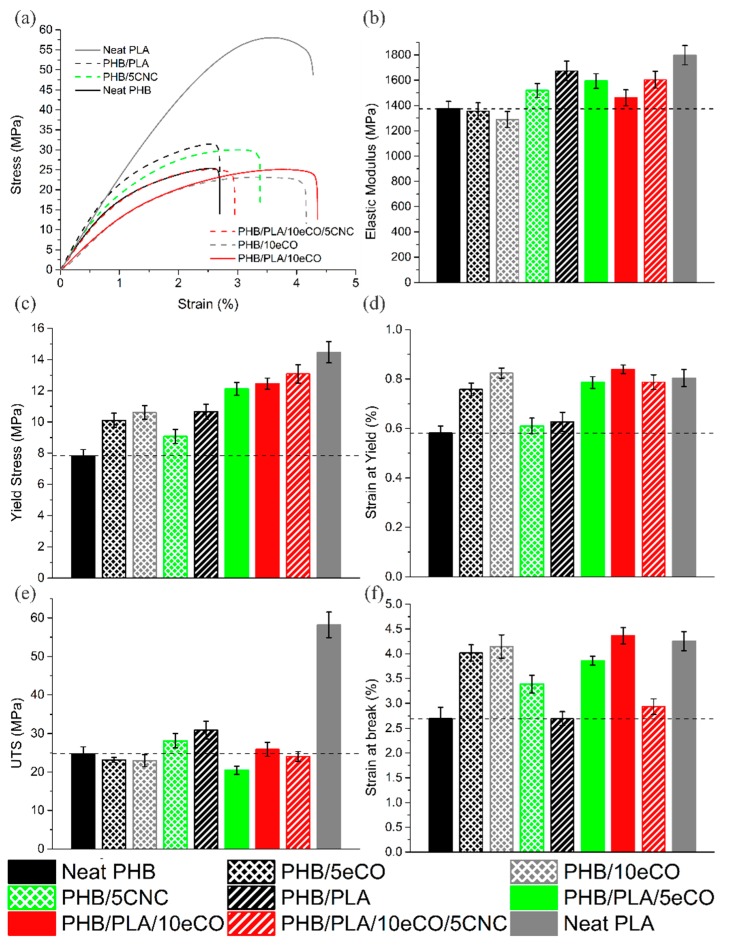
(**a**) Stress-strain curves as well as (**b**) *E,* (**c**) yield stress, (**d**) strain at yield, (**e**) σ_UTS_, and (**f**) ε_b_ values for neat polyhydroxybutyrate (PHB), neat poly(lactic acid) (PLA) and seven eCO/CNC plasticized PHB samples.

Representative stress-strain curves of the neat PHB, PHB/10eCO, PHB/5CNC, PHB/PLA, PHB/PLA/10eCO, PHB/PLA/10eCO/5CNC, and neat PLA compositions are shown in Figure 2a. The stress-strain curves of all compositions consistently presented elastic and plastic regions, as reported for PHB, PLA, and their blends [9,22]. The values of Young’s modulus (*E*), yield stress (σ_y_), strain at yield (ε_y_), ultimate tensile stress (σ_UTS_), and strain at break (ε_b_) are reported in Figure 2b–f for all compositions described in Table 1. The properties of neat PHB were found to be *E* = 1374 MPa ± 58 MPa, σ_y_ = 7.83 MPa ± 0.4 MPa, ε_y_ = 0.58% ± 0.03%, σ_UTS_ = 24.76 MPa ± 1.79 MPa and ε_b_ = 2.69% ± 0.23%. These results, aligning with those reported by Seoane et al. [12] and Anbukarasu et al. [5], provide evidence of the brittle behaviour of PHB particularly due to the low strain at break [12,16]. Other studies have presented higher mechanical properties (*E* = 3500 MPa, σ_UTS_ = 40 MPa, ε_b_ = 8%) than those presented in this work [40,41,42]. A source for this discrepancy between studies may be that as result of a biological process, PHB has been shown to present a wide range of physical properties; this includes mechanical properties as well as density and molecular weight [40,41,42]. 

In order to increase the strain at break of PHB, epoxidized canola oil (eCO) was added to the polymer matrix [13]. With the addition of 5 wt % eCO (i.e., PHB/5eCO) to neat PHB, an increase in ε_b_ from 2.69% ± 0.23% to 4.01% ± 0.17% was observed, and a similar value (ε_b_ = 4.15% ± 0.24%) was observed with 10 wt % eCO in PHB. Similar effects were found in ε_y_: the addition of 5 wt % and 10 wt % eCO to neat PHB led to small but statistically significant increases in ε_y_, from 0.58% ± 0.03% (neat PHB) to 0.76% ± 0.03% and 0.82% ± 0.02%, respectively. These changes may occur because the plasticizers lowered the amount of secondary bonds between the polymer chains, reducing the overall interactions between polymer molecules, and increasing the free volume, mobility and flexibility of the chains [13]. However, no reduction in *E* (which would normally be expected for a plasticized material [13]) was observed.

Given that, contrary to expectations, a significant drop in *E* did not occur with the addition of eCO, PLA was added in the hope of overcoming this phenomenon. The addition of 25 wt % PLA to neat PHB led to a large increase in *E* (from 1374 MPa ± 58 MPa to 1673 MPa ± 77 MPa), σ_y_ (7.83 MPa ± 0.4 MPa to 10.65 MPa ± 0.49 MPa), and σ_UTS_ (25 MPa ± 2 MPa to 31 MPa ± 2 MPa), as shown in Figure 2b,c,e. Despite these increases, there was no change in the ε_b_ (2.69% ± 0.23% for neat PHB and 2.7% ± 0.14% for PHB/PLA). This suggests that PLA may be working as a reinforcement phase with higher mechanical properties than the PHB matrix, thereby improving its strength and stiffness.

We explored whether the rule of mixtures could be used to predict the mechanical properties of further blends of PHB and PLA. The rule of mixtures, $K_{B}=V_{1}K_{1}+V_{2}K_{2}$, gives an estimated value for a given property, *K_B_*, often of a composite (in this case a polymer blend). This property is estimated based on the properties of the constituents in bulk (*K_1_* and *K_2_*), where *V_1_* and *V_2_,* are their respective volume fraction in the blend [43,44]. As the densities of PHB and PLA are approximately equal (1.2 g/cm^3^ [45]), the volume fraction of the blend is the same as the mass fraction: in this case 25% PLA and 75% PHB. Given the volume fraction and the mechanical properties reported above, the rule of mixture would lead to *E* = 1478 MPa, σ_y_ = 10.65 MPa, and σ_UTS_ = 49.47 MPa. These estimations have been found to be within 11.6%, 10.9%, and 15% margins of error, respectively, which are acceptable for a rough estimation of future polymer blends close to 25 wt % content. 

The study of synergistic effects of PLA and eCO on mechanical properties was done by characterizing the properties of PHB/PLA/5eCO, PHB/PLA/10eCO, and PHB/PLA/10eCO/5CNC. Similar to the case of neat PHB, the addition of 5 wt % eCO to the PHB/PLA blend led to an increase in ε_y_ (from 0.63% ± 0.04% for PHB/PLA to 0.79% ± 0.02% for PHB/PLA/5eCO) and in ε_b_ (from 2.69% ± 0.14% for PHB/PLA to 3.86% ± 0.09% for PHB/PLA/5eCO). In addition, when 10 wt % eCO was added to the PHB/PLA blend, ε_y_ increased (from 0.63% ± 0.04% for PHB/PLA to 0.84% ± 0.02% for PHB/PLA/10eCO), as did ε_b_ (from 2.69% ± 0.14% for PHB/PLA to 4.37% ± 0.17% for PHB/PLA/10eCO). These results suggest that the role of the plasticizer in increasing the strain at yield and strain at break of the polymers by increasing the free volume, mobility and flexibility of the chains was consistent between neat PHB and PHB/PLA blends [13]. Similar to the effect seen in neat PHB, the average *E* and σ_UTS_ decreased upon the addition of 10 wt % eCO (from 1673 MPa ± 77 MPa to 1461 MPa ± 63 MPa, and 31 MPa ± 2 MPa to 26 MPa ± 2 MPa, respectively), when compared to the PHB/PLA samples.

The effect of adding CNCs to neat PHB was determined. The addition of 5 wt % CNCs into the PHB matrix (i.e., PHB/5CNC) led to increases in *E*, σ_y,_ and σ_UTS_ (from 1374 MPa ± 58 MPa to 1518 MPa ± 55 MPa, from 7.83 MPa ± 0.4 MPa to 9.07 MPa ± 0.45 MPa, and from 24.8 MPa ± 1.8 MPa to 28.1 MPa ± 1.9 MPa, respectively). In addition, ε_b_ increased from 2.69% ± 0.23% to 3.39% ± 0.18%. This improvement in mechanical properties has been reported to be linked to behaviour associated with the efficient dispersion of CNCs, which allows for higher interfacial adhesion between them and the matrix [22,46].

The combined effect of all additives on mechanical properties was studied in the composition PHB/PLA/10eCO/5CNC. This composition can be most easily compared with PHB/PLA/10eCO, which exhibited higher modulus, yield stress, ultimate tensile stress, and strain at break than neat PHB. However, the addition of 5 wt % CNCs into the PHB/PLA/10eCO sample did not significantly change the yield stress (average σ_y_ of 12.45 MPa ± 0.36 MPa for PHB/PLA/10eCO and 13.08 MPa ± 0.58 MPa for PHB/PLA/10eCO/5CNC) or the strain at yield (average ε_y_ of 0.84% ± 0.02% and 0.79% ± 0.03% for the PHB/PLA/10eCO and PHB/PLA/10eCO/5CNC compositions, respectively). Although the addition of CNCs is generally expected to result in an increase in Young’s modulus, when added to the PHB/PLA/10eCO blends, the Young’s modulus did not undergo a statistically significant increase. This addition of CNCs into the matrix did however reduce the average σ_UTS_ and ε_b_ (from 26 MPa ± 2 MPa to 24 MPa ± 1 MPa and 4.37% ± 0.17% and 2.93% ± 0.16%, respectively) while providing an expected increase in *E* (from 1461 MPa ± 63 MPa to 1604 MPa ± 66 MPa). These results help conclude that the CNCs were not as effective as reinforcing agents as expected. Moreover, previous work showed the simultaneous increase in *E* and ε_b_ with the addition of nanocrystals into a PLA/PHB matrix in which PLA is the dominant component (71.25 wt %) [22]. It is possible that the CNCs formed clusters within the predominant PHB matrix rather than dispersing evenly in both phases; this would lead to the observed reduction in strain at break as these points may act as points of stress initiation. Alternatively, these results may be indicative of poor interfacial adhesion between the CNCs and matrix in the presence of the oil. 

**Figure 3:**
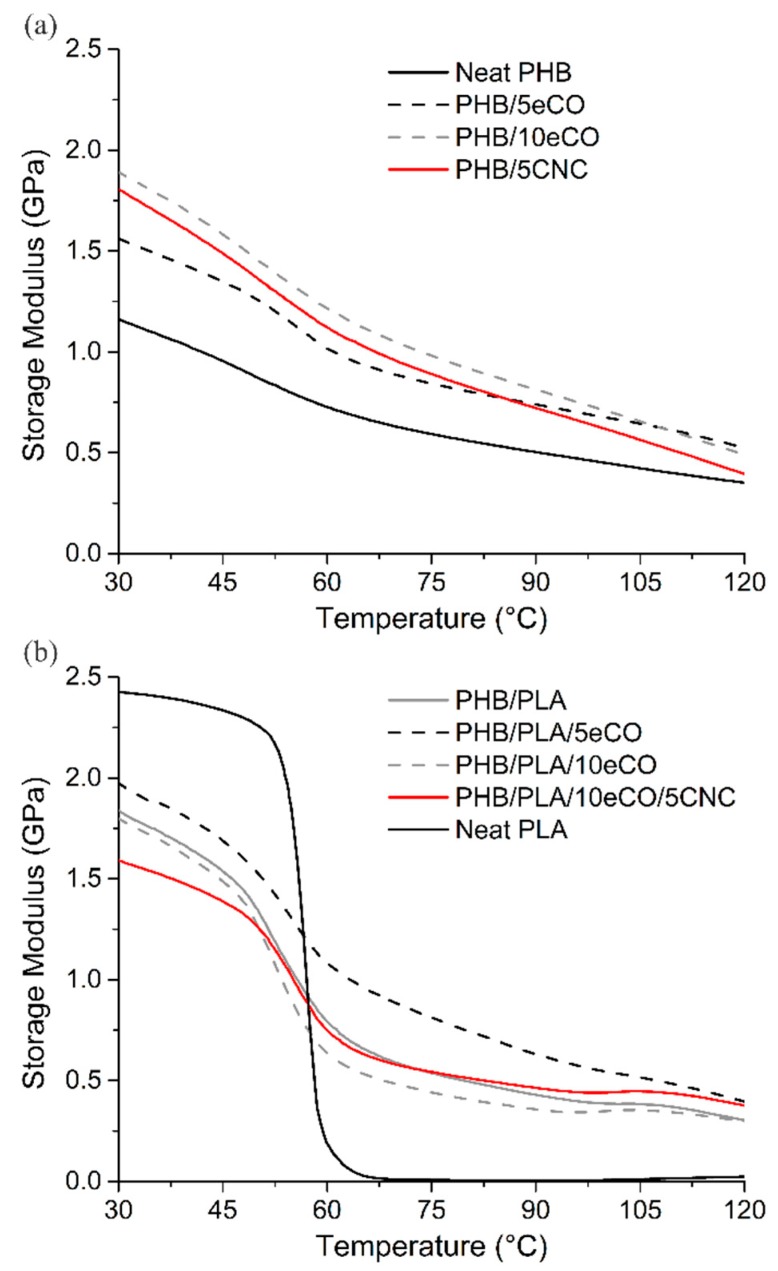
Behavior of *E*’ over temperature of (**a**) polyhydroxybutyrate (PHB)-based, and (**b**) PHB/PLA-based polymer blends.

For comparison, single cantilever mode dynamic mechanical analysis (DMA) was used to characterize the mechanical properties of the composites under tension/compression and shear stresses [47]. The results for the tested samples are shown in Figure 3. In Figure 3a, the influence of each additive (i.e., eCO, CNCs and PLA) on the storage modulus (*E*’), the energy stored by the material through deformation, is shown. The storage modulus for neat PHB at 30 °C was 1.16 GPa, while with the additives, the values increased to 1.56 GPa, 1.89 GPa, and 1.80 GPa for 5 wt % eCO, 10 wt % eCO and 5 wt % CNCs, respectively. 

The effect of adding 25 wt % PLA to PHB on the storage modulus was also studied. When PLA was blended with PHB, the storage modulus increased from 1.16 GPa to 1.84 GPa. This represents a 58% relative increase in *E’* with respect to neat PHB. As is shown in Figure 3b, the storage modulus of the neat PLA is higher than neat PHB, with an intense drop around 60 °C, which corresponds the *T*_g_ of PLA. This drop in storage modulus was consistently present in those compositions containing PLA.

In addition, eCO in concentrations of 5 and 10 wt % eCO and a combination of 10 wt % eCO plus 5 wt % CNCs (15 wt % of filler) were added to the PHB/PLA blend, enhancing the storage modulus at 30 °C from 1.16 GPa (neat PHB) to 1.97, 1.80 and 1.59 GPa, respectively.

While the values for storage modulus do not match those from the tensile tests, they follow similar trends in the effect that additives have in the elastic properties of the composites. One reason for these discrepancies may be linked to the difference in load modes between tensile test (uniaxial normal stress) and single cantilever (normal and shear stresses). 

### 3.3. Crystallinity and Thermal Stability

**Figure 4:**
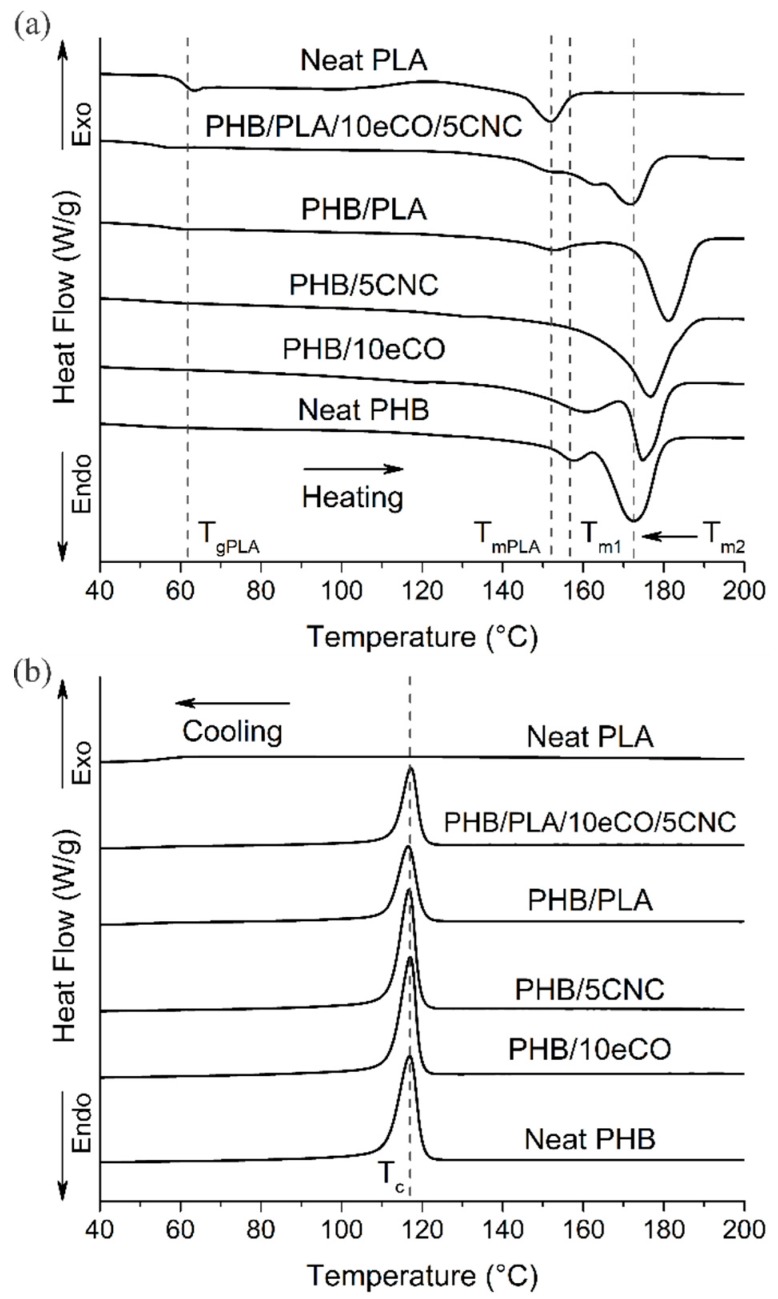
DSC (**a**) Heating and (**b**) cooling cycles curves for different polyhydroxybutyrate (PHB), poly(lactic acid) (PLA), and PHB/PLA polymer composites (aged 7 days).

The crystallinity and thermal properties of PHB composites were evaluated using DSC and TGA, respectively. Figure 4 shows representative DSC heating (Figure 4a) and cooling (Figure 4b) curves while Table 2 summarizes the thermal properties and crystallinity. During the heating process (Figure 4a), neat PHB samples presented a double melting peak at *T*_m1_: 157 °C ± 2 °C, and T_m2_: 172 °C ± 1 °C. The double peak during melting of PHB is common. Initially, metastable crystals—incomplete crystals formed during processing—are melted in the first peak while stable crystals are melted at a higher temperature (corresponding to the second peak) [5,9,12,13,48]. During the cooling process, a crystallization peak was found at approximately 108 °C ± 2 °C, with no variation observed with the blending of PLA, eCO, or CNCs. The cooling cycle of PLA did not present any crystallization event due to the low crystallinity of this polymer. While the melting and crystallization events of PHB were consistent throughout all compositions, those containing PLA showed a distinct glass transition, *Tg* (also observed during the cooling cycle), and melting, T_mPLA_, at approximately 60 °C and 151 °C, respectively (Figure 4a), corresponding to this second polymer. These peaks indicate that the materials are not completely miscible. 

The crystallinity of the PHB phase in neat polymer and blended composites was calculated using Equation (1) and summarized in Table 2, as measured after one day and seven days. The enthalpy of melting was estimated by the integration of the melting peaks of the DSC curve, excluding the melting peak of PLA (Figure 4a). On day 1 after preparation, the crystallinity of both neat PHB and the PHB phase in PHB/PLA was very similar (20.5% ± 1.6% and 22.9% ± 2.1%). However, after seven days of aging, the crystallinity of neat PHB increased to 28.2% ± 1.5%, while that of the PHB/PLA blend increased only slightly (to 23.5% ± 1.8%). These results suggest that the addition of PLA suppresses the gradual crystallization of PHB at room temperature (which is above the glass transition temperature of PHB, ~5 °C [5]). This decrease in crystallinity of PHB with the blending of PLA has been previously reported [9,25,46].

In parallel to the effect of PLA, the changes in crystallinity of PHB with the addition of eCO and CNCs were studied. As for the PHB/eCO samples, the crystallinity of all composites containing eCO was approximately 20% after 1 day. However, after seven days, the crystallinity of samples containing 5 wt % and 10 wt % eCO increased significantly to 34.0% ± 1.2% for PHB/5eCO and 37.1% ± 1.6% for PHB/10eCO, while the crystallinity of PHB/PLA/5eCO and PHB/PLA/10eCO increased to 41.8% ± 1.0% and 42.4% ± 1.8%, respectively. The addition of the plasticizer eCO appears to significantly increase the chain mobility of PHB polymer chains, allowing them to form crystals during the ageing process at room temperature. A similar increase in crystallinity has been observed previously in PHB utilizing plasticizers such as glyceryl tributyrate [12].

As proposed above, the addition of PLA may suppress the crystallization of PHB at room temperature. This could have a detrimental effect on the elastic properties of the PHB phase [22]. A way of mitigating this drop in crystallinity could be the addition of CNCs [19]. In this case, the addition of 5 wt % CNCs led to a significant increase in the crystallinity of neat PHB, even after one day (26.5% for PHB/5CNC vs. 20.5% for PHB alone), suggesting that the CNCs act as nucleating sites during thermal processing and cooling. Crystallization continued during aging, and after seven days the crystallinity reached 39.6% ± 1.6% for PHB/5CNC (compared with 28.2% for PHB alone). Several previous studies have also shown that the addition of nanoparticles such as nanoclay [49], montmorillonite (MMT) [50], titanium oxide (TiO_2_) [51], and CNCs into PHB/PLA blends and PHBV/PLA blends increased the crystallinity of the polymer matrix.

The inclusion of both eCO and CNCs in a blend of PHB/PLA did not result in further increases in crystallinity. Interestingly, after 1 day the crystallinity of the PHB/PLA/10eCO/5CNC sample was low, indicating that, unlike for the PHB/5CNC sample, the CNCs could not act as an effective nucleating agent for PHB in this complex system, possibly segregating preferentially in the PLA phase. After seven days, PHB/PLA/10eCO samples with and without CNCs had crystallinities of 43.4% ± 2.5% and 42.4% ± 2.1%, respectively, suggesting that the eCO was still able to enhance the mobility of the PHB chains during aging.

**Figure 5:**
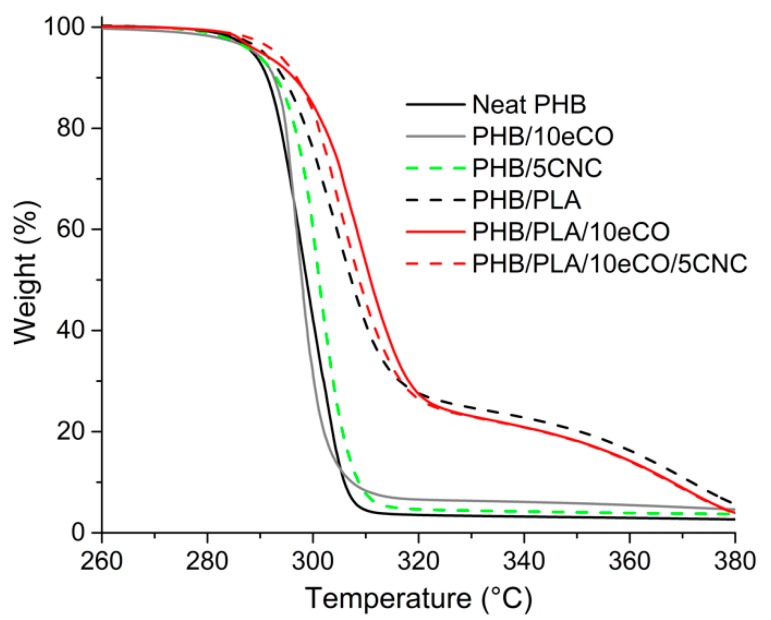
TGA curves showing the influence of additives on weight loss for polyhydroxybutyrate (PHB) and PHB/PLA composites.

The thermal stability of PHB-based blends was studied using TGA. Figure 5 shows the resulting curves for all the samples. The TGA results suggest that all compositions were stable at onset temperatures as low as 281 °C ± 4 °C. Here, as in other studies, it was found that the degradation temperature of neat PHB was about 290 °C ± 5 °C [4,5,12,52]. In general, the addition of other materials to the PHB matrix, (i.e., blending of PLA, eCO, or CNC) did not result in a significant change in thermal stability of the material. The average onset degradation temperature (defined as the intersection of the tangents of the curve before the drop in sample weight and that of the highest degradation rate) was 290 °C ± 5 °C. This suggests that while PLA and eCO degrade at higher temperatures than PHB (340 °C and 360 °C, respectively), the use of these additives did not affect the mechanisms of thermal degradation of PHB. Similar behaviour has been reported in the past [49,53]. Figure 5 shows that compositions containing PLA presented initial degradation at 290 °C ± 5 °C, likely related to PHB, and a second stage, at around 335 °C ± 15 °C, related to PLA and eCO. A comparison of the rate of thermal degradation of compositions with and without PLA revealed that the addition of this polymer into PHB significantly decreased the rate of degradation of the composite. The rate of thermal degradation (calculated as the derivative of weight with respect to temperature) of those compositions without PLA was 9.47 wt %/°C while that of compositions containing PLA was 3.54 wt %/°C. These TGA results show that no significant change in the thermal stability of the matrix was found.

## 4. Discussion

The first objective of this work was to determine whether eCO could be used as a plasticizer for PHB. As previously reported by Özgür et al. [13] among others [12,54], the addition of a plasticizer is expected to lead to a decrease in *E* and an increase in the strain at break. However, the tensile test and DMA results did not show a significant change in *E* with the addition of eCO (Figure 2). This likely results from the fact that the addition of the plasticizer additionally resulted in an increase in the crystallinity of the samples after 7 days of aging at room temperature—from 28.2% for neat PHB to 34.0% and 37.1% for PHB/5eCO and PHB/10eCO, respectively. Samples with higher crystallinity are also expected to have more stiffness, thereby mitigating the effect of the plasticizer on the chains under mechanical deformation. This effect was in fact observed as the modulus of the PHB/eCO samples was only slightly lower than the modulus of the pure PHB despite the inclusion of the plasticizer (Figure 2). Moreover, a statistically significant increase in the elongation at break was seen with the addition of eCO to the PHB samples, although the final value was still relatively low (4.15% ± 0.24%) as compared with conventional polymers. The change in elongation at break was observed as a difference between the failure surface of neat PHB and PHB/eCO (Figure 1). While smooth surfaces, characteristic of brittle failure, were observed for neat PHB (Figure 1a), signs of plastic deformation were seen in images of samples containing eCO (Figure 1b), suggesting the plasticizer did modify the mechanical behavior of the material despite the limited change in modulus. This also suggests that the plastic deformation of the amorphous regions of the material was enhanced despite the overall increase in crystallinity. To reduce the overall crystallinity of the PHB, the amorphous polymer PLA was blended with this material in a 3:1 PHB to PLA ratio. Interestingly, the addition of PLA to the semi-crystalline PHB impeded aging of the material, with low crystallinity values of approximately 22% observed at both day 1 and day 7 (whereas all other samples underwent significant changes in crystallinity by day 7). The stiffness (reflected by *E*) and toughness (reflected by σ_y_, and σ_UTS_) of the blends were higher than those of pure PHB, suggesting PLA works as a reinforcing agent within the PHB. The *E*, σ_y_, and σ_UTS_ of the blends were calculated using the rule of mixtures, and the measured and calculated values differed by a maximum of 15%. However, the strain at break of the PHB:PLA samples was the same as for pure PHB (2.7% ± 0.23%), despite the fact that pure PLA exhibits a strain at break of 4.26% ± 0.19%. The low strain at break of the blends likely resulted from the limited miscibility of the polymers, which was reflected by the distinct glass transition and melting peaks of PLA in the DSC data (Table 2) and by the fibrous structures observed in the SEM images of fractured samples previously characterized by SEM (Figure 1).

The strain at break of the PHB:PLA blends could be improved by the addition of the plasticizer eCO. While the resulting strain at break of the PHB/PLA/10eCO (4.37% ± 0.17%) and of the PHB/10eCO sample (4.15% ± 0.24%) were similar, other mechanical properties of these samples differed. For instance, the reduction in *E* observed with the addition of eCO to neat PHB was mitigated by the PLA in the blend (E of PHB/10eCO = 1289 MPa ± 63 MPa, *E* of PHB/PLA/10eCO = 1461 MPa ± 62 MPa). Similarly, relatively high values of σ_y_ and σ_UTS_ were observed for the PHB/PLA/10eCO blend. This composition presented the best combination of mechanical properties tested in this work. Interestingly, the PHB/PLA/10eCO blend also exhibited the second highest crystallinity after 7 days aging (only supplanted by PHB/PLA/10eCO/5CNC). This indicates that while the amorphous fraction of PHB in this material is relatively low, these materials are still able to accommodate relatively high strain at break.

To further explore the relationship between crystallinity and mechanical properties, and to see if high Young’s modulus could be achieved, 5 wt % CNCs was added to both neat PHB and PHB/PLA/10eCO. CNCs have been shown to increase the crystallinity of the polymer matrix as they work as nucleation sites for crystals [22,46]. The PHB/5CNC sample exhibited the highest crystallinity at 1 day of any of the samples (26%), suggesting that the CNCs acted as nucleating agents during the initial processing of the materials. In contrast, the PHB/PLA/10eCO/5CNC sample exhibited the same initial crystallinity (~20%) as the rest of the samples, showing that the inclusion of the eCO and/or PLA inhibits the ability of the CNCs to nucleate PHB crystals. With the addition of 5 wt % of CNCs into the PHB matrix (i.e., PHB/5CNC), an increase in *E*, σ_y,_ and σ_UTS_ was found. This increase in properties has been reported to be associated with the efficient dispersion of CNCs, which allows for a high interfacial adhesion between them and the matrix. In agreement with these results, Bhardwaj et al. [10,29] have reported an increase in *E* (from 1321 MPa to 1412 MPa) and σ_UTS_ (from 36 MPa ± 1.4 MPa to 45 MPa ± 1.8 MPa) with a 5 wt % addition of CNCs. In addition, their work concludes that concentrations as low as 2 wt % of CNCs in the PHB matrix could lead to higher improvements in mechanical properties as this allows for better dispersion and intercalation reinforcement into the PHB matrix. Given that this effective dispersion of CNCs has been repeatedly linked with the increase in mechanical properties, the results presented in this work suggest that CNCs were fairly dispersed within the PHB matrix [10,22,29,49].

In contrast to the effects of adding CNCs to neat PHB, when CNCs were added to the PHB/PLA/10eCO materials, there was little change in the strength or stiffness of the material. The only mechanical property that underwent a large change was the elongation at break, which in fact decreased from 4.37% ± 0.17% (PHB/PLA/10eCO) to 2.93% ± 0.16% (PHB/PLA/10eCO/5CNC). In this complex system, interactions amongst the components impede the expected individual effect of CNCs in PHB. For example, epoxy groups prevalent in the eCO may react with the hydroxyl groups of the CNCs, effectively passivating the surface of the CNCs. 

## 5. Conclusions

This work aimed to improve the mechanical properties of PHB by blending it with eCO, CNCs and PLA, and to establish whether these additives affect the thermal stability of this biopolymer. We found the addition of PLA and CNCs led to an increase in Young’s modulus (*E*), yield stress (σ_y_), strain at yield (ε_y_), ultimate tensile stress (σ_UTS_), and storage modulus (*E’*) of neat PHB. In parallel, the addition of eCO in concentrations of 5 wt % and 10 wt % led to an increase in elongation at break. In addition, the enhanced mobility of the polymer chains resulting from the presence of eCO, coupled with an ageing time of seven days at room temperature, led to an increase in the crystallinity of the PHB phase in the composites. This increase in crystallinity was found to increase the storage modulus. The results in this study demonstrated that the combination of more than one or all components (e.g., PHB/PLA/10eCO) led to synergistic effects on mechanical and thermal performance. Overall, the addition of PLA as reinforcing phase and of eCO as plasticizer led to an increase in tensile strength, strain at break, and thermal stability of the composite compared to neat PHB. We demonstrate the synergetic use of PLA and eCO increases the elastic properties and strain at break of PHB while avoiding the decrease in thermal stability of the composites. In addition to this, we compared how different strategies for increasing the mechanical properties of PHB (blending with other polymers, adding plasticizers and CNCs) work to tailor the mechanical performance of PHB. Moreover, studying the impact strength of these materials may provide relevant information for the development of new applications with PHB and PHB/PLA blends and composites. While this is not included in the scope of this work, the authors suggest that an investigation of other properties, such as hardness perhaps by means of Rockwell E hardness test or impact strength by Charpy testing, should be conducted in the future.

We demonstrate that, similar to previous studies by Zhang et al. [9] on PLA, Arrieta et al. [22] on CNCs, and Seoane et al. [12] and Garcia-Garcia et al. [16] on epoxidized vegetable oils, the inclusion of additives in PHB can tailor the properties of this fully biodegradable polymer. The results presented herein could lead to an increased value for canola oil in regions where this natural resource is highly available. In particular, the study of synergistic effects of the additives in this work can present opportunities for the development of sustainable biodegradable polymers that can compete with current petroleum-based plastics, and which release environmentally benign materials upon degradation.

## Figures and Tables

**Table 1:** Sample names and compositions of blends used in this work.

Sample Name	PHB:PLA (wt:wt)	eCO wt %	CNCs wt %
Neat PHB	1:0	0	0
PHB/5eCO	1:0	5	0
PHB/10eCO	1:0	10	0
PHB/5CNC	1:0	0	5
PHB/PLA	3:1	0	0
PHB/PLA/5eCO	3:1	5	0
PHB/PLA/10eCO	3:1	10	0
PHB/PLA/10eCO/5CNC	3:1	10	5
Neat PLA	0:1	0	0

**Table 2:** Thermal properties and crystallinity of polyhydroxybutyrate (PHB) and PHB/PLA blends by DSC (as measured after seven days, unless otherwise indicated).

Composition	T_c_^1^ (°C)	T_m1_^1^ (°C)	T_m2_^1^ (°C)	Xc,1 Day^2^ (%)	Xc,7 Days^2^ (%)
Neat PHB	117	157	172	20.5	28.2
PHB/5eCO	117	158	173	21.4	34.0
PHB/10eCO	116	161	175	20.8	37.1
PHB/5CNC	117	-	176	26.4	39.6
PHB/PLA	116	-	181	22.9	23.5
PHB/PLA/5eCO	116	159	171	19.2	41.8
PHB/PLA/10eCO	115	161	170	20.7	42.4
PHB/PLA/5CNC/10eCO	115	161	171	20.3	43.4
Neat PLA^3^	-	151	-	1.4	1.2
1: Max standard deviation = 3 °C. 2: Max standard deviation = 2.5%. 3: Crystallinity of neat PLA with $\Delta H_{m}^{0}=96\;J/g$ [39]					

## Supplementary Materials

The following are available online at https://www.mdpi.com/2073-4360/11/6/933/s1, Supplementary Information: Influence of epoxidized canola oil (eCO) and cellulose nanocrystals (CNCs) on the mechanical and thermal properties of polyhydroxybutyrate (PHB)—poly(lactic acid) (PLA) blends.
