# Supplementary material for: Influence of Epoxidized Canola Oil (eCO) and Cellulose Nanocrystals (CNCs) on the Mechanical and Thermal Properties of Polyhydroxybutyrate (PHB)—Poly(lactic acid) (PLA) Blends
Source: Polymers (Basel). 2019 May 29;11(6):933. doi: 10.3390/polym11060933 (PMC6631437; doi:10.3390/polym11060933)
Supplement: Supplementary file 1 [file polymers-11-00933-s001.pdf]

polymers

MDPI

Supplementary Information

Supplementary Information: Influence of epoxidized canola oil (eCO) and cellulose nanocrystals (CNCs) on the mechanical and thermal properties of polyhydroxybutyrate (PHB) – poly(lactic acid) (PLA) blends

Adrián Lopera-Valle [1], Joseph Caputo [1], Rosineide Leão [2], Dominic Sauvageau [1], Sandra Maria Luz [1,2], and Anastasia Elias [1,*]

[1] Department of Chemical and Materials Engineering, University of Alberta, Donadeo Innovation Centre for Engineering, Edmonton, Alberta, T6G 1H9, Canada
[2] Department of Automotive Engineering, University of Brasília, Faculdade do Gama, Brasília - DF, 72444-240, Brazil
* Correspondence: aelias@ualberta.ca;

1. Gel Permeation Chromatography (GPC)

The molecular weight of polyhydroxybutyrate (PHB) was evaluated using gel permeation chromatography (GPC). The GPC instrument was equipped with a column (PAS-106M, 300 × 8 mm, 10 µm, PolyAnalytik Inc., London, Canada), a Liquid Chromatography pump and autosampler (Viscotek GPCmax, Malvern Panalytical, Malvern, UK), a refractive index detector (Viscotek TDA302, Malvern Panalytical, Malvern, UK). The molecular weight of the samples was determined based on calibration curves obtained using a polystyrene standard. Chloroform ($CHCl_3$) was used as eluent at a constant flow of 1 ml/min. The PHB specimens (n = 3) were dissolved using $CHCl_3$ (specimen concentration of 0.3 mg/ml), and filtered (at 60°C through a 45 µm syringe filter) before injection. The molecular weight of PHB was found to be Mw: 190 kDa.

2. Fourier Transform Infrared Spectroscopy (FTIR)

FTIR spectra of the canola oil before and after epoxidation were recorded using a Fourier Transform Infrared Spectrometer (Agilent Cary 600 Series FTIR Spectrometer, Agilent Technologies Inc., Santa Clara, CA, USA) instrument equipped with a universal attenuated total reflectance (UATR) accessory. The spectra were recorded between 4000 cm^{-1} and 400 cm^{-1}.

The FTIR spectra of the canola oil was recorded in order to characterize whether the epoxidation reaction occurred. Figure S1 illustrated the canola oil spectra before and after epoxidation. As reported by previous studies [1, 2], the FTIR peak at 825 cm^{-1} corresponds to the epoxy group that was incorporated into the canola oil after the epoxidation process. Other relevant peaks are illustrated in Figure S1.

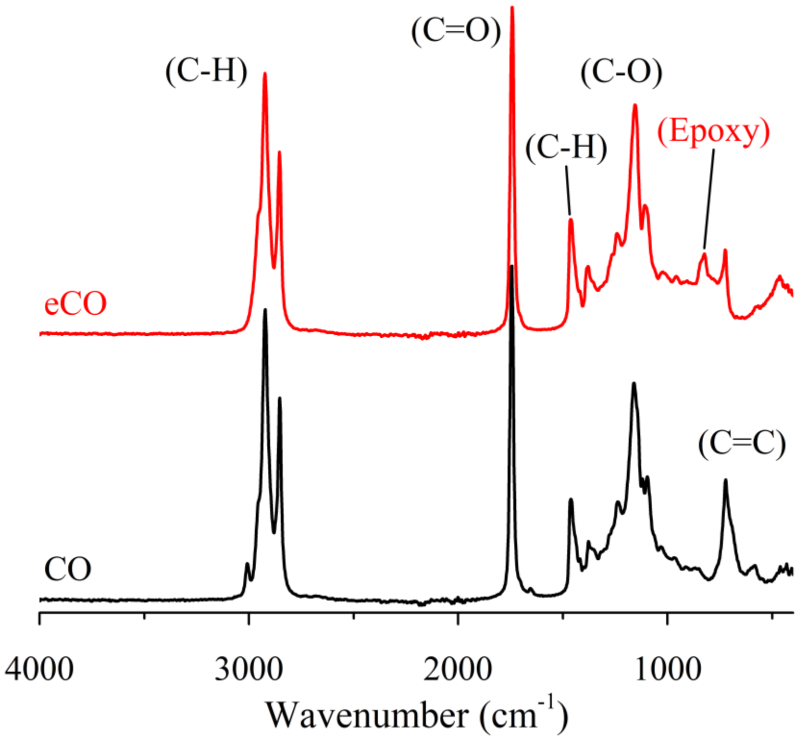

Figure S1: FTIR curves between (a) 4000 cm^{-1} and 400 cm^{-1} of canola oil (CO) and epoxidized canola oil (eCO) samples

3. Transmission electron microscopy (TEM)

The sample preparation for TEM was done by deposition a drop of CNC aqueous suspension onto a carbon microgrid (300 mesh) and allowing it to dry. A 1 % solution of uranyl acetate was used to stain the carbon grid. The samples was observed using a transmission electron microscope (TEM JEM 2100, JOEL Ltd., Peabody, MA, USA) operating at 80 keV. Figure S2 shows representative TEM images of the CNCs samples. The length and diameter was found to be 220 nm and 12 nm, respectively.

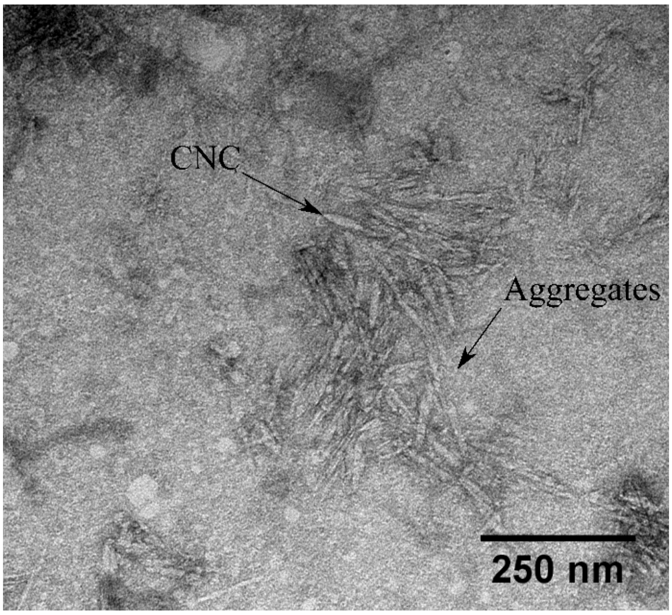

Figure S2: Representative TEM image of individual and aggregated cellulose nanocrystals from sugarcane bagasse

4. X-Ray diffraction

The crystallinity of the CNCs was estimated using a X-ray diffractometer (XRD, Ultima IV, Rikagu Co., Woodlands, TX, USA) eqioped with a Cu K_α filament tube (wavelength of 1.54 Å). A step width of 0.05°/min in the 2θ axis was set between 5° and 50°. The energizing voltage was 40 kV at a current of 40 mA. The crystallinity was calculated based on the Segal's equation (Eq. S1) using the intensity of the 200 (at 2θ = 22.6°) and 110 (at 2θ = 15.5°) peaks [3, 4]. These peaks represent the crystalline and amorphous phases of the CNCs.

$$x_{CNC} = \frac{I_{200} - I_{100}}{I_{100}} \qquad \text{(Eq. S1)}$$

References

1. Mungroo, R.; Pradhan, N. C.; Goud, V. V.; Dalai, A. K. Epoxidation of Canola Oil with Hydrogen Peroxide Catalyzed by Acidic Ion Exchange Resin, J. Am. Oil Chem. Soc., 2008, vol. 85, no. 9, pp. 887–896. DOI: 10.1007/s11746-008-1277-z.
2. Chieng, B. W.; Ibrahim, N. A.; Then, Y. Y.; Loo, Y. Y. Epoxidized Vegetable Oils Plasticized Poly(lactic acid) Biocomposites: Mechanical, Thermal and Morphology Properties, Molecules, 2014, vol. 19, no. 10, pp. 16024–16038. DOI: 10.3390/molecules191016024.
3. Segal, L.; Creely, J. J.; Martin, A. E.; Conrad, C. M. An Empirical Method for Estimating the Degree of Crystallinity of Native Cellulose Using the X-Ray Diffractometer, Text. Res. J., 1959, vol. 29, no. 10, pp. 786–794. DOI: 10.1177/004051755902901003.
4. Leão, R. M.; Miléo, P. C.; Maia, J. M. L. L.; Luz, S. M. Environmental and technical feasibility of cellulose nanocrystal manufacturing from sugarcane bagasse, Carbohydr. Polym., 2017, vol. 175, no. November, pp. 518–529. DOI: 10.1016/j.carbpol.2017.07.087.

© 2019 by the authors. Submitted for possible open access publication under the terms and conditions of the Creative Commons Attribution (CC BY) license (http://creativecommons.org/licenses/by/4.0/).
